# A new method for safety helmet detection based on convolutional neural network

**DOI:** 10.1371/journal.pone.0292970

**Published:** 2023-10-13

**Authors:** YueJing Qian, Bo Wang

**Affiliations:** 1 Zhejiang Industry and Trade Vocational College, Wenzhou, Zhejiang, China; 2 Zhejiang College of Security Technology, Wenzhou, Zhejiang, China; Sichuan University, CHINA

## Abstract

Considering practical issues such as cost control of hardware facilities in engineering projects, it is a challenge to design a robust safety helmet detection method, which can be implemented on mobile or embedded devices with limited computing power. This paper presents an approach to optimize the BottleneckCSP structure in the YOLOv5 backbone network, which can greatly reduce the complexity of the model without changing the size of the network input and output. To eliminate the information loss caused by upsampling and enhance the semantic information of the feature map on the reverse path, this paper designs an upsampling feature enhancement module. Besides, To avoid the negative impact of redundant information generated by feature fusion on the detection results, this paper introduces a self-attention mechanism. That is, using the designed channel attention module and location attention module, adjacent shallow feature maps and upsampled feature maps are adaptively fused to generate new feature maps with strong semantics and precise location information. Compared with the existing methods with the fastest inference speed, under the same compute capability, the proposed method not only has faster inference speed, the FPS can reach 416, but also has better performance with mAP of 94.2%.

## 1. Introduction

Construction sites usually have many hidden safety hazards, which are prone to safety accidents such as people injury caused by falling objects and heavy objects. In particular, in construction and production operations, accidents occur every year, due to the face that many workers do not properly wear safety helmets. Safety helmets are an important protective tool for workers on construction sites, but many workers choose not to wear safety helmets due to the lack of comfort, which will lead to danger to workers’ lives [1]. At present, the supervision wearing safety helmets is mostly done manually, which requires a lot of human intervention, is thus costive and subject to subjective errors. Therefore, it is desirable to apply machine vision for automatic safety helmet wearing detection. This prevents the occurrence of safety accidents to a certain extent and ensures the safety of workers. With the continuous development of artificial intelligence and other technologies, machine vision has become a commonly used method in engineering, which also provides new ideas for automatic detection of safety helmet wearing. Traditional machine vision methods usually use hand-crafted features, i.e. haar features, for safety helmet detection [2]. However, in real construction sites, the performance of traditional algorithms is not satisfactory due to the variations of illumination, viewing angle, and occlusion.

In recent years, with the rapid development of deep learning technology, Convolutional Neural Networks (CNN) has been widely used in object detection tasks. The typical algorithms mainly include the YOLO [3,4] series proposed by Redmon et al., and the single-stage algorithm of the SSD [5,6] series proposed by Liu et al. These algorithms tend to detect faster and is more suitable for practical applications. The classic two-stage method Faster R-CNN [7] proposed by Ren et al., firstly generates a series of candidate object regions, and then classifies and regresses the bounding boxes. Though the accuracy of two-stage approaches are higher, their detection speed is relatively slow. Moreover, convolutional neural networks often require a huge amount of computation and parameters, thus usually rely on hardware with strong computing power, such as GPU, to complete the training and inference process. However, high-performance computing hardware is often not available in construction sites [8]. Therefore, designing a lightweight safety helmet detection model is very important for the actual construction engineering management.

To obtain a better helmet detection effect on hardware devices with limited computing power, this paper proposes a new method for helmet detection based on CNN, denoted as SHDet. SHDet uses YOLOv5 as the backbone and reconstructs the feature extraction module to reduce the computational complexity of the detection network. And, the performance of the detection model is improved by the designed upsampling module and attention module. Overall, our technical contributions in this work mainly include the following three points.

(1) Our work improved the Inverted Resblock (IR) structure proposed by G. Howard et al, and successfully integrated the structure into the BottleneckCSP structure of the YOLOv5 backbone network, which can greatly reduce the complexity of the model without changing the size of the network input and output.

(2) To eliminate the information loss caused by upsampling and enhance the semantic information of feature maps on the reverse path, this paper designs an upsampling feature enhancement module. This module combines subpixel convolution and layer-by-layer parallel dilated convolution to expand the resolution and receptive field of feature maps. Therefore, the context information of the object can be better utilized, and the sensitivity of the network to small objects can be enhanced.

(3) To mitigate the negative impact of redundant information generated by feature fusion on the detection results, this paper proposes an adaptive feature fusion module, which applies a concatenated channel attention module and positional attention module to pay more attention on key information. This enables the model to adaptively fuse adjacent shallow feature maps and upsampled feature maps.

## 2. Related work

Safety helmet detection is the foundation for monitoring the safety production of construction sites and can provide typical applications for enterprise intelligent video surveillance. In recent years, there have been numerous studies on safety helmet detection, and we generally categorize these detection methods into two types: traditional machine learning-based methods and deep learning-based methods.

While detection methods based on traditional machine learning have certain limitations, they have also made some progress. Specifically, Rattapoom Waranusast et al. [9] first localize moving objects and extract features from this region. They then use a K-nearest neighbor classifier to classify them as motorcycles or other moving objects, and based on the projected contours, they count and segment the heads of riders on motorcycles. Finally, they determine whether the heads are wearing helmets. Furthermore, Wu et al. [10] proposed a color-based hybrid descriptor comprising local binary patterns, Hu-moment invariants, and color histograms to extract features for helmets of different colors. Subsequently, a hierarchical support vector machine is constructed to classify all features into four categories, achieving an average accuracy of 90.3%. Romuere Silva et al. [11] introduced a hybrid descriptor based on local binary patterns, histograms of oriented gradients, and Hough transform descriptors for automatically detecting motorcyclists without helmets. Similarly, Romuere Rodrigues et al. [12] employed circular Hough transform and oriented gradient histogram descriptors to extract image attributes. They then utilized a multi-layer perceptron classifier for detecting helmetless motorcyclists.

To extract more effective safety helmet features, several safety helmet detection methods based on HOG and SVM have been proposed. For instance, Rubaiyat et al. [13], combined frequency domain information of images with a human detection algorithm to detect construction workers. They subsequently applied color and circular Hough transform-based feature extraction techniques to detect helmets. Li et al. [14] first employed the ViBe background modeling algorithm to detect moving objects, and extracted histogram of oriented gradients (HOG) features from the resulting motion regions to describe individuals within them. Based on the HOG feature extraction results, they trained a support vector machine to classify pedestrians, ultimately identifying whether a helmet is worn or not based on color features. Jin et al. [15] initially utilized the deformable part model to locate workers, followed by color feature matching to extract potential helmet regions. Finally, safety helmet detection is carried out using an SVM trained with HOG features.

With the rapid advancement of deep learning in recent years, many methods based on convolutional neural networks (CNNs) have also achieved promising results in safety helmet detection [16]. Currently, two-stage detection methods based on Faster RCNN and one-stage detection methods based on SSD, RetinaNet, and YOLO are widely used for safety helmet detection.

Although two-stage object detection methods offer higher detection accuracy, they often require more inference time. For instance, Gu et al. [17,18] introduced an advanced deep learning-based approach to determine whether a worker is wearing a safety helmet. This approach first employs multi-scale training and anchor points to enhance the robustness of the original Faster RCNN algorithm against scale variations. Then, an online hard sample mining method is employed to optimize the model and alleviate the imbalance between positive and negative samples. Finally, the geometric information of the detected object is used to determine whether the worker is wearing a safety helmet. Similarly, Chen et al. [19] proposed an enhanced Faster R-CNN algorithm for helmet detection. The algorithm incorporates Retinex image enhancement technology to improve image quality in complex outdoor scenes and applies the K-means++ clustering algorithm to better adapt to small-scale safety helmets.

While one-stage object detection methods lead to improved inference time, they also trade off detection accuracy. For example, addressing the low accuracy and poor robustness of traditional detection methods, Dai et al. [20] proposed enhancements to the SSD (Single Shot MultiBox Detector) object detection network. First, sensitivity to small object detection is enhanced by fusing multi-layer low-level and high-level semantic features. Second, a lightweight network structure is designed to reduce model parameters and computation. Targeting the low accuracy of existing helmet detection methods, Guang et al. [21] introduced an SSD-based object detection algorithm. The algorithm employs spatial attention mechanisms for low-level features and channel attention mechanisms for high-level features. A feature pyramid and multi-scale perception module are introduced to enhance robustness to scale variations. Furthermore, an effective adaptive anchor box adjustment method is designed, allowing adaptive adjustment of the scale distribution of anchor boxes on each feature map layer according to feature map size. Experimental results demonstrate that the detection model achieves 88.1% and 80.5% mean average precision (mAP) on helmet and VOC 2007 datasets, respectively. Additionally, for better detection of overlapping small objects, Ai et al. [22] introduced a layer-wise feature weighting module after feature maps of different scales to generate a score matrix of the same size as the feature map. Pointwise multiplication is then applied between the score matrix and feature map to filter out irrelevant noise. The experiment shows that, compared to the original RetinaNet with ResNet50 backbone, although mAP is improved by 4.19%, model forward inference time remains relatively long.

Due to its speed advantage, the YOLO series method has been widely applied in the industrial field. For instance, Sun et al. [23] proposed an improved object detection algorithm based on YOLOv3, which incorporates attention mechanisms to enhance the expressive capability of feature maps within the neural network, thereby enhancing the robustness of the detection model. Additionally, the authors redesigned the loss function to address the imbalance of positive and negative samples. Experimental results indicate that the new algorithm enhances average accuracy by 6.4% compared to the previous algorithm. Huang et al. [24] employed an improved YOLOv3 algorithm to output the location information of the target object, followed by pixel feature statistics to predict the confidence level of the helmet. Subsequently, workers are assessed for helmet usage based on empirical thresholds.

Furthermore, to balance the accuracy and speed of the safety helmet detection model, several safety helmet detection methods based on YOLOv4 and YOLOv5 have been proposed. For instance, Deng et al. [25] introduced a helmet detection method based on an enhanced YOLOv4. The author employed the K-means algorithm to acquire prior box centers and more targeted edge information. Subsequently, a multi-scale training strategy was adopted during network training to enhance the model’s adaptability to different object scales. Experimental results reveal that the mean Average Precision (mAP) value of this method reaches 92.89%, with a detection speed of 15 FPS. Gu et al. [26] addressed the challenges of detecting helmets under complex postures of construction workers by proposing a helmet detection method based on attitude estimation. In the pose estimation model of OpenPose, the author introduced a residual network to optimize feature extraction and better identify the head area of construction workers. Whether a worker is wearing a helmet is determined by the intersection of the head region with the helmet region. Based on the YOLOv5 approach, Tan et al. [27] incorporated a target scale detection function to enable the model to better adapt to small targets. Subsequently, DloU-NMS was employed instead of NMS to achieve more accurate suppression of neighboring bounding boxes. Kun et al. [28] added a fourth detection scale to YOLOv5 to detect smaller objects and introduced an attention mechanism in the backbone network to construct more informative features. Experimental results demonstrate that the model achieves an average accuracy of 92.2%, with an inference time of 3.0 milliseconds for a 640×640 image.

## 3. Methods

**Fig 1:**
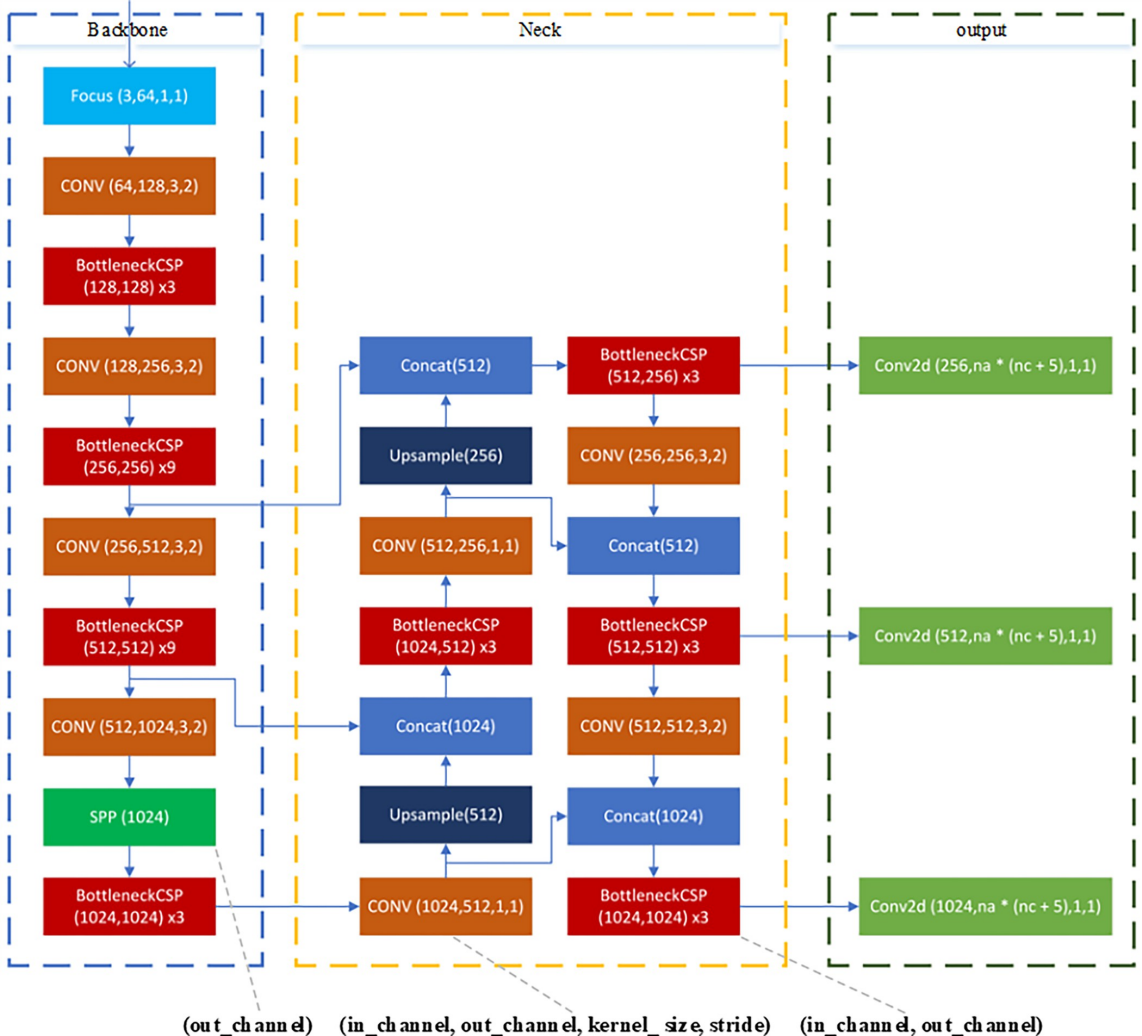
The network structure diagram of the original YOLOv5.

YOLOv5 is a one-stage method that does not have the process of extracting candidate boxes from images, but unifies object detection as a regression problem. That is, the entire image is used as the input of the network, and the position and category of the objects are directly regressed at the output layer. Its network structure is shown in Fig 1.

Mosaic data augmentation is used in YOLOv5 training. Four images are randomly selected and spliced after scaling and color perturbation to increase the number of small targets during training, thereby improving the model’s ability to detect small targets. On the backbone network, YOLOv5 mainly adopts the Focus structure and the CSPnet structure [29] to improve the feature extraction capability and speed up the inference speed of the detection model. Moreover, YOLOv5 uses GIOU_Loss [30] to effectively solves the problem of non-overlapping bounding boxes. Based on the above ideas, YOLOv5 has achieved better results in detection accuracy and detection speed.

**Fig 2:**
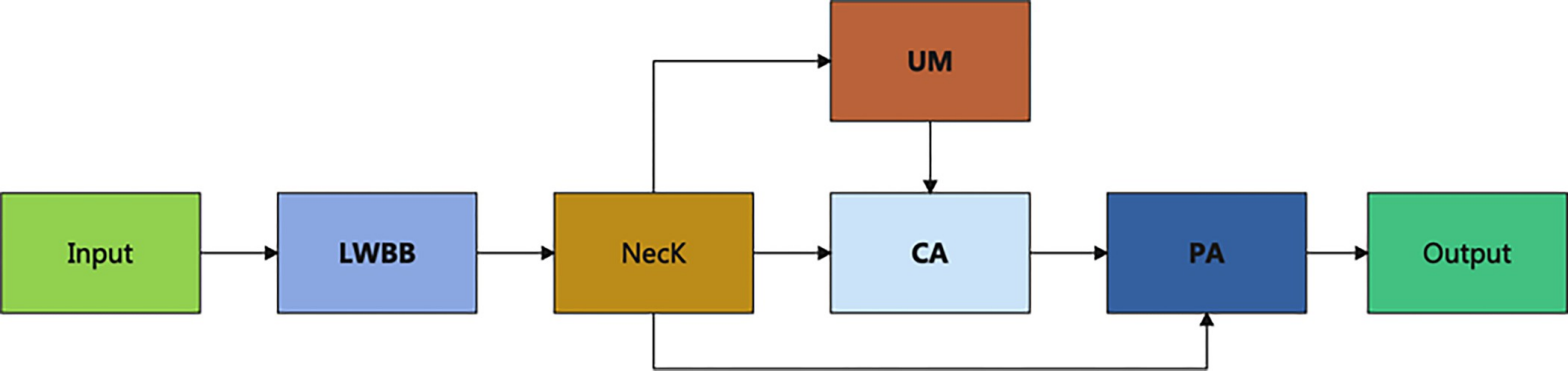
The network architecture diagram of the proposed YOLOv5-based SHDet method. Abbreviations: LWBB—LightWeight BackBone, UM—Upsampling Module, CA—Channel Attention, PA—Positional Attention.

In order to balance the speed and accuracy of the detection model, this study optimized the Backbone module of YOLOv5 to reduce computational complexity. Additionally, following the Neck module, an upsampling module capable of enhancing features and an attention module capable of eliminating redundant information were designed to enhance the model’s detection accuracy. The network architecture diagram of the proposed SHDet method based on YOLOv5 is illustrated in Fig 2. Among them, LWBB stands for the designed Lightweight Backbone module; UM represents the designed Upsample Module used for feature enhancement; CA represents the designed Channel Attention mechanism aimed at eliminating redundancy within channels; PA denotes the designed Positional Attention mechanism used to remove spatial redundancy. Furthermore, the input of UM is derived from the output of the Neck, the input of CA comes from the outputs of both the Neck and UM, and the input of PA originates from the outputs of both the Neck and CA.

### 3.1 Lightweight backbone

**Fig 3:**
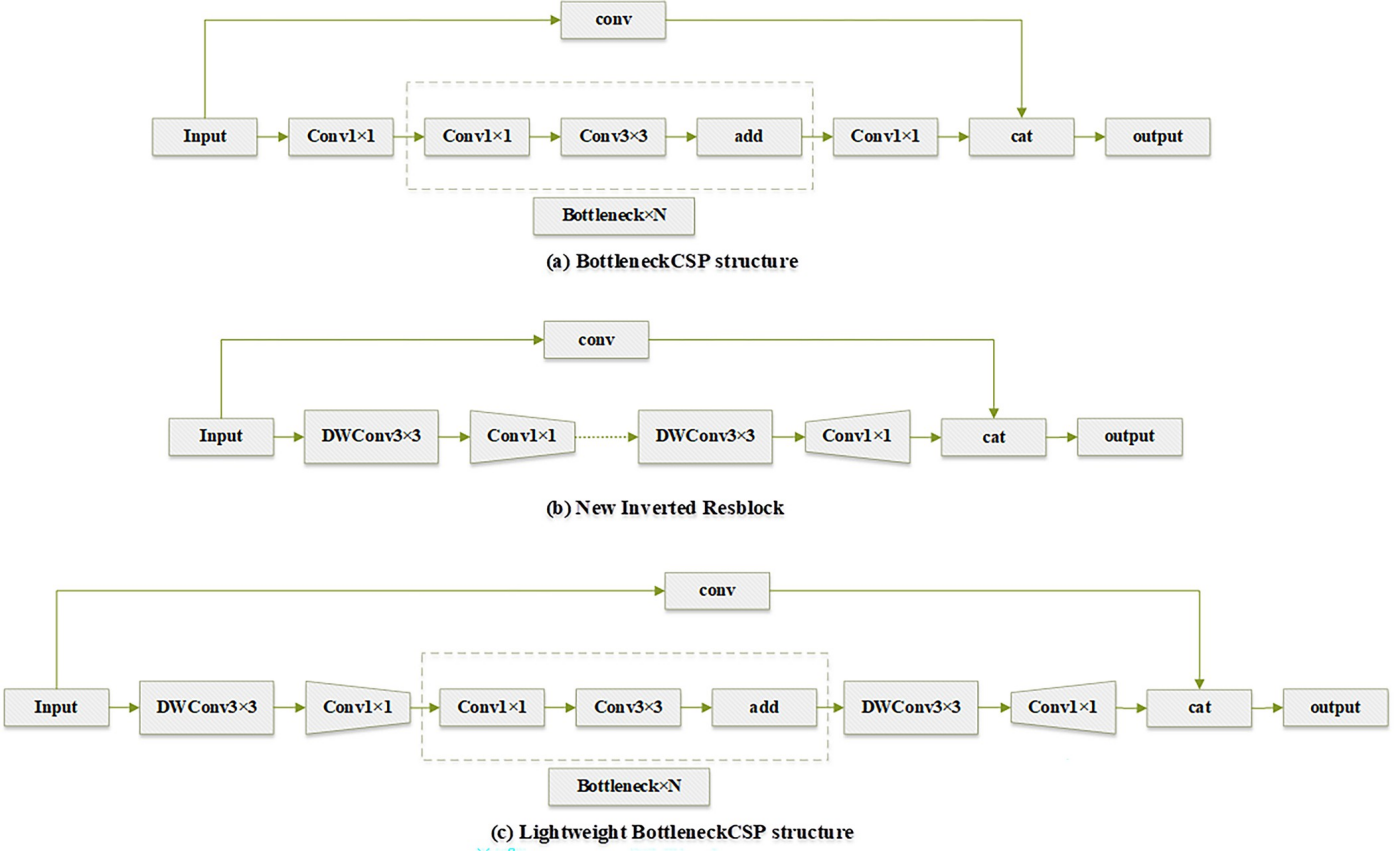
The designed lightweight BottleneckCSP structure is shown in Fig 3(C), the new IR structure is shown in Fig 3(B), and the original BottleneckCSP structure is shown in Fig 3(A).

The BottleneckCSP structure is the main component of the YOLOv5 backbone network, and is also the part with the largest amount of computation. The corresponding network structure diagram is shown in Fig 3(A). To reduce the number of parameters of this structure, this paper introduced the IR structure proposed by Andrew G. Howard et al [31] into this module. In IR structure, the 1×1 convolution is used to increase the dimension first, then the 3×3 depth separable convolution DWConv is used to reduce the amount of calculation parameters, and then the 1×1 convolution is used to reduce the dimension, and finally the result is connected to the input. However, Daquan et al., [32] found that IR structure has gradient confusion, and 1×1 convolution reduces spatial information. Therefore, according to the problems in IR structure and residual block, we reconstructed a new IR structure, which allows more spatial information to be passed into the neural network, so that the depthwise separable convolution can extract richer features. The network structure diagram is shown in Fig 3(B).

In particular, because IR structure has gradient confusion, we reuse the residual block. As 1×1 convolution will reduce the spatial information, we add a 3×3 DWConv after the input to get more spatial information. In response to the problem of the residual block, we set the activation function in the first DWConv and the last 1×1 convolution to construct a new IR structure. Finally, it is integrated into the BottleneckCSP structure, to get a lightweight BottleneckCSP structure. The designed lightweight BottleneckCSP structure first performs a 3×3 DWConv, and then uses a 1×1 convolution to reduce the dimension. After the dimension reduction, a 3×3 DWConv is performed, and then the 1×1 convolution is used to increase the dimension again, and finally concatenated together with the input. The corresponding network structure diagram is shown in Fig 3(C).

### 3.2 Upsampling module

As the resolutions of feature maps with different scales are different, the small size feature map needs to be upsampled before feature fusion. Most of the commonly used upsampling methods enlarge the image size by interpolation or deconvolution, which may lead to the loss of effective semantic information of feature maps. Different from traditional upsampling methods, subpixel convolution [33] enlarges the resolution of feature maps, while reducing the number of channels of feature maps by rearranging and combining different channels. Therefore, it reduces the loss of feature map information and saves more effective information. The mathematical definition of subpixel convolution is shown in Formula (1).

$${SC(I)}_{r∙H,r∙W,C}=I_{H,W,C∙r∙r}$$ (1)

In Formula (1), the symbol SC signifies the operation of transforming the feature map dimensions from H×W×Cr^2^ to rH×rW×C. Here, H, W, and C denote the height, width, and number of channels of the feature map, respectively. The variable r stands for the scaling factor, while I represents the input feature map.

The up-sampled feature map will inevitably lose some feature information. Using dilated convolution [34] to expand the receptive field can extract the context information of the object, thereby enhancing the semantic information of the feature map. Receptive fields of different sizes can detect targets of different scales. A smaller receptive field is beneficial to detect small targets, and a larger receptive field can extract a wider range of context information. However, the dilated convolution cannot sample the hole part, which will cause the extracted information to be non-continuous. Therefore, this module uses a layer-by-layer parallel method to concatenate together dilated convolutions with different dilation rates to extract the feature information of continuous holes. Moreover, the dilated convolution with larger receptive field can also help extract the context information around the small object, thus improve the detection accuracy of the small object.

To eliminate the information loss caused by upsampling, we enhance the semantic information of the feature maps on the reverse path, and design an upsampling feature enhancement module. This module combines sub-pixel convolution and layer-by-layer parallel dilated convolution to expand the resolution and receptive field of feature maps, and enhance the network’s sensitivity to small objects by utilizing the contextual information of the object. The module is divided into three parts: subpixel convolutional layer, feature enhancement layer and connection layer (Concat). Among them, the sub-pixel convolution layer uses sub-pixel convolution to perform upsampling operations, adjust the size of the feature map, and reduce the number of feature map channels. The feature enhancement layer consists of three layer-by-layer parallel dilated convolutions (DConv), and the dilation rates of the dilated convolutions are 1, 3, and 5, respectively. The dilated convolution with dilation rate of 1 and 3 is suitable for detecting the location information of small objects, and the dilated convolution with dilation rate of 5 can provide richer contextual information for small objects. Dilated convolutions with different receptive fields are connected in parallel layer by layer, and multi-scale feature information can be exchanged between them to enhance the continuity of feature information. The connection layer connects the feature maps output with the feature enhancement layer in a cascaded manner, and retains the feature information of different receptive field feature maps.

**Fig 4:**
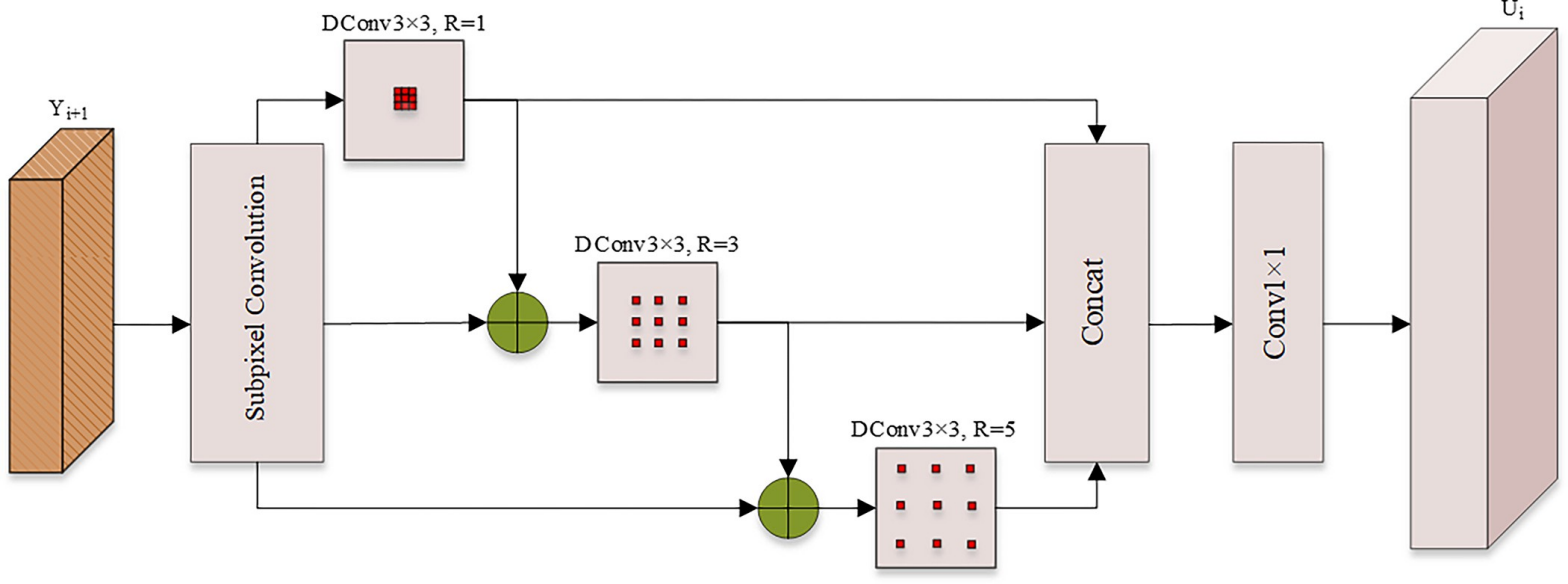
Network structure diagram of the upsampling module based on subpixel convolution and dilated convolution.

The network structure of the upsampling module is shown in Fig 4. First, the input layer Y_i+1_ is input to the sub-pixel convolution layer to double the resolution of the feature map while reducing the number of channels to a quarter of the original. Then, the up-sampled feature map is divided into three branches, and sent to the dilated convolution layers with expansion rates of 1, 3, and 5 in parallel layer by layer, so as to obtain three feature maps with different receptive field sizes. Finally, the three feature maps are aggregated by splicing the number of channels. The number of channels of the feature layer is changed by 1×1 convolution, so that the number of channels of U_i_ is the same as that of the input, but with doubled resolution. Among them, Y_i+1_ refers to the i+1th prediction layer from shallow to deep of the Neck module of YOLOv5, and U_i_ refers to the up-sampled output layer corresponding to Y_i+1_.

### 3.3 Channel attention

The feature maps fused by element-by-element addition are easy to generate redundant information. If only the context information of the feature map is extracted by global average pooling, and the weights of different channels are recalibrated, the key features and background features in the same channel might be equally weighted, such that the characteristics of small objects are weankened. Therefore, this paper proposes a channel attention module to aggregate two different spatial context information by parallel global average pooling and global max pooling, which can enhance useful features and reduce redundant information introduced by upsampling feature maps.

**Fig 5:**
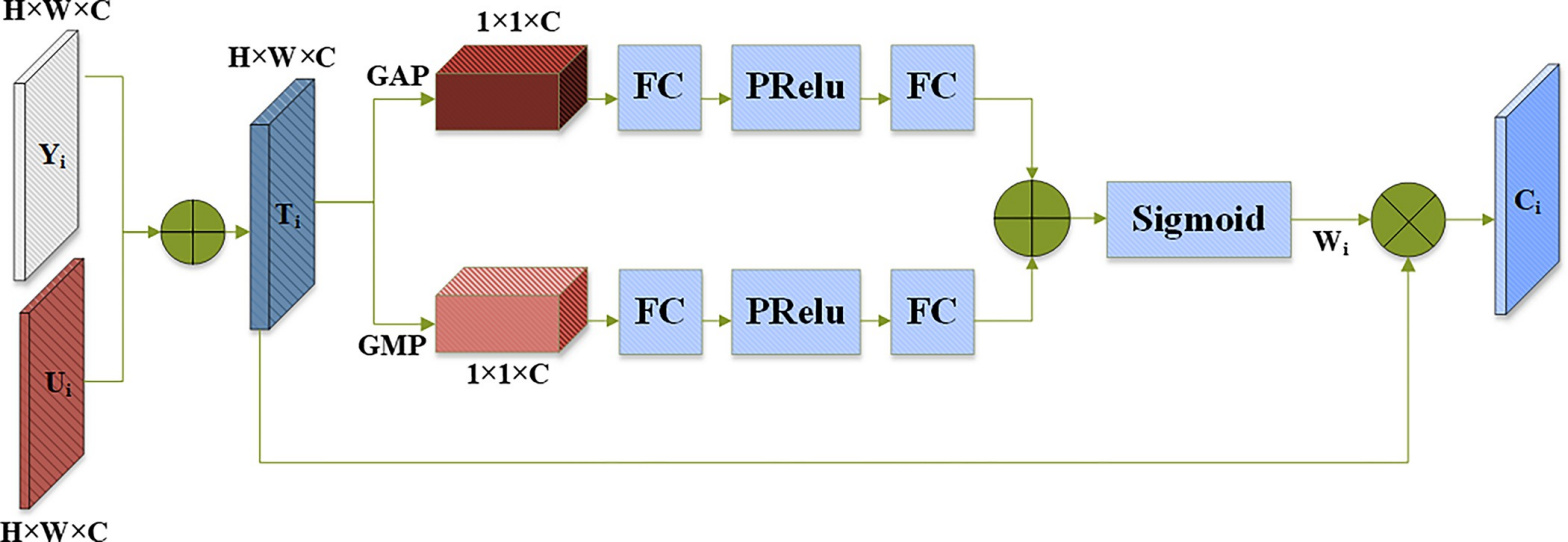
Channel attention module structure.

The designed channel attention structure is shown in Fig 5. First, Y_i_ and U_i_ are added element by element to obtain the feature map T_i_∈R^H×W×C^. Among them, Y_i_ is the ith prediction layer from shallow to deep of the Neck module, and U_i_ is the output layer of the ith upsampling module. Second, two feature maps P_i1_ and P_i2_ containing different spatial context information are generated using global average pooling GAPool and global max pooling GMPool. Among them, {P_il_, P_i2_}∈R^1×1×C^, can be expressed as:

$$P_{i1}=GAPool(T_{i})$$ (2)

$$P_{i2}=GMPool(T_{i})$$ (3)

Subsequently, the feature information is passed to the fully connected layer and the PRelu activation layer [35], respectively. The two fully connected layers are used to reduce and increase the number of channels, respectively, and the PRelu activation layer learns the nonlinear relationship between different channels. Then, our approach aggregate the information of two different channels, and use the activation function (Sigmoid) to generate a set of weights for each channel, which reflect the correlation between channels and can be expressed by Formula (4):

$$W_{i}=Sig(F(PR(F(P_{i1})))+F(PR(F(P_{i2}))))$$ (4)

Where, Sig represents the sigmoid function, F represents the fully connected layer and PR represents the activation layer function.

Finally, the feature map T_i_ and the channel weight W_i_ are multiplied element by element to obtain the channel attention feature map C_i_. This process can be expressed by Formula (5):

$$C_{i}=W_{i}\times T_{i}$$ (5)

### 3.4 Positional attention

The channel attention module identifies and focuses on more meaningful channels in the feature map, and does not consider which parts of the feature map are more important. Therefore, this paper designs a self-attention mechanism-based spatial attention module that utilizes the attention information of shallow and deep feature maps to highlight the importance of object spatial regions. This module aims to learn the association between features at any two locations and enhance the expressive ability of the features at key spatial locations.

**Fig 6:**
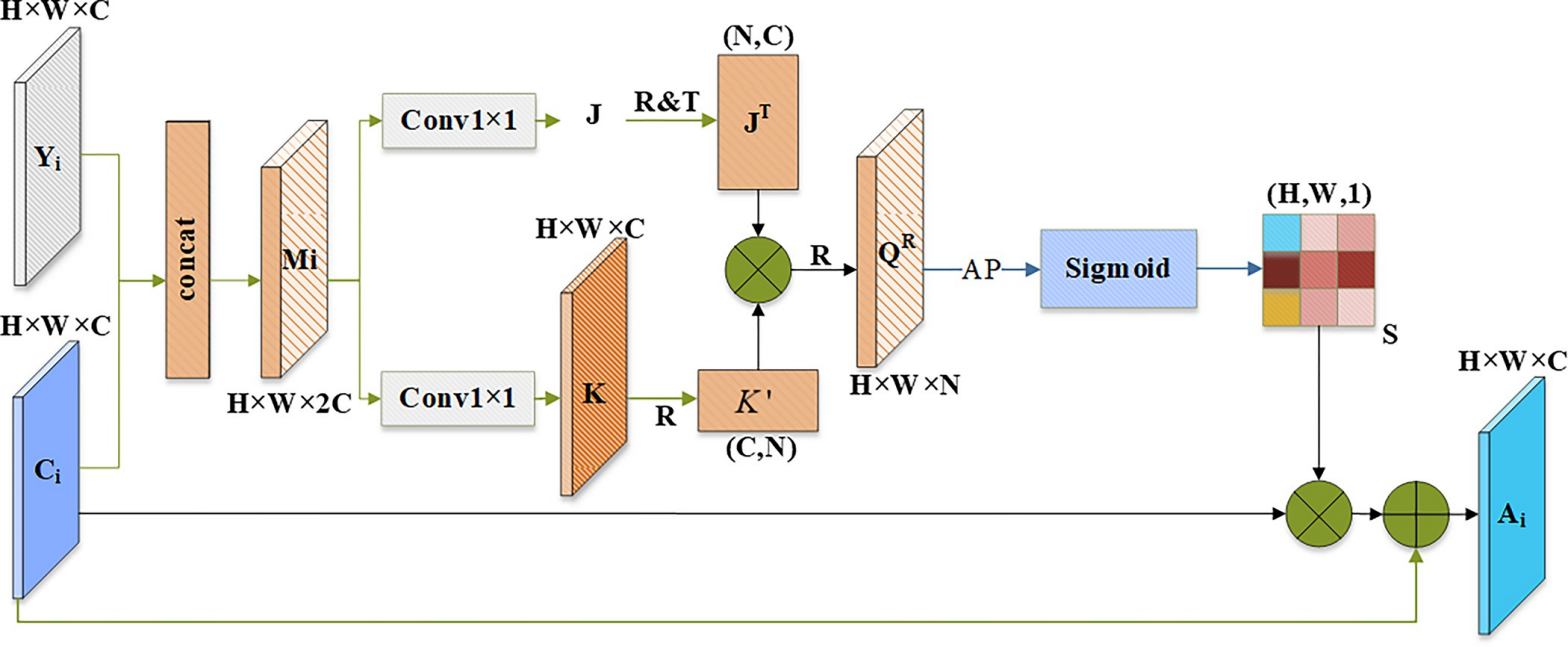
Positional attention module structure.

The designed positional attention structure is shown in Fig 6. First, the shallow feature map Y_i_ and the channel attention feature map C_i_ are cascaded, and the number of channels of the feature map is merged to generate the feature map M_i_∈R^H×W×2C^. And through two parallel 1×1 convolutions with BN layer and ReLU layer, two feature maps J and K are generated, {J,K}∈R^H×W×C^. Then, we convert J to matrix J^T^∈R^N×C^ by reshape (R) and transpose (T) operations, and convert K to matrix K′∈R^C×N^ by Reshape operation, where N = H×W. After that, matrix multiplication of J^T^ and K′ is performed to generate a correlation matrixQ∈R^N×N^. We Reshape Q to convert it into a feature map Q^R^∈R^H×W×N^, and then use Average Pooling (AP) and sigmoid activation function to get the attention matrix S∈R^H×W×1^. Finally, the generated attention matrix S is multiplied by C_i_ pixel by pixel and then added element by element to obtain the location attention feature map.

The above process can be expressed using Formula (6–Formula (8) as below.


$$Q=J^{T}\times K\prime$$ (6)

$$S=Sig(AP(Q^{R}))$$ (7)

$$A_{i}=S⊗C_{i}⊕C_{i}$$ (8)

## 4. Experimental results and discussion

To verify the feasibility and practicability of the lightweight helmet detection network proposed in this paper, this section introduces the dataset, the evaluation indicators, and the experimental results and analysis.

In training, the optimizer used in this paper is the SGD (Stochastic Gradient Descent) optimizer [36], the batch size is set to 32, the initial learning rate is 0.001, the momentum is set to 0.937, the weight decay is 0.0005, and the training algebra is 300.

### 4.1 Data and implementation details

We gathered a collection of images featuring helmets and individuals across diverse scenarios, encompassing the Internet, substations, and construction sites. In total, 6,116 images were amassed, each adorned with a rectangular frame and a category label for the helmets, following the PASCAL VOC format. Furthermore, alongside categorizing the helmets, we also annotated the corresponding head regions. Specifically, in instances where workers were not donning safety helmets, we annotated their head regions and assigned the "head" category. It’s noteworthy that our annotations were exclusively directed at safety helmets worn by construction site workers; helmets worn by pedestrians in everyday contexts remained unannotated. Our self-curated dataset of safety helmets was christened as SCSH. Within this paper, the images within the dataset underwent a random partition into training, validation, and testing subsets, adhering to an 8:1:1 ratio. The training, validation, and testing subsets encompass 4,892, 612, and 612 images, respectively. In our pursuit of diversifying the dataset, we employed data augmentation techniques on the training subset, encompassing rotations of 45 degrees and brightness reduction (alphas = 0.26). This augmentation expanded the training subset’s scale threefold, culminating in a grand total of 14,676 images.

In order to assess the effectiveness of our proposed method across diverse scenarios, we also employed the publicly accessible Safety Helmet Wearing Dataset (SHWD). This dataset serves as a valuable resource for both safety helmet detection and human head recognition. The SHWD encompasses images annotated with "helmet" and "head" categories, accompanied by bounding box labels, all adhering to the PASCAL VOC format. A grand total of 7,581 images are included in this dataset. Similarly to our approach, the SHWD dataset was randomly partitioned into training, validation, and testing subsets, maintaining an 8:1:1 distribution ratio.

We conducted our experiments on two high-performance computers. Their hardware specifications are as follows: one is equipped with an Nvidia GeForce GTX1080Ti GPU and an AMD Ryzen 5 4600H CPU running at 3.0 GHz, while the other features an Nvidia GeForce RTX2080Ti GPU and an AMD Ryzen 9 5900HX CPU operating at 3.3 GHz. Our experiments were conducted on the Ubuntu 18.04 operating system, utilizing the PyTorch deep learning framework and the Python 3.7 programming language for model training.

### 4.2 Evaluation criteria

The evaluation metric used in this paper is multi-class mean average precision (mAP). The higher the mAP, the better the performance of the model. To observe whether the proposed SHDet helmet detection method meets the real-time requirements, we use FPS (Frames Per Second) to characterize the inference speed of the model.

The calculation formulas of mAP are shown in Formulas (9)–(12).

$$mAP=\frac{\sum_{i}^{N}{AP}_{i}}{N}$$ (9)

$$AP=\int_{0}^{1}p(r)dr$$ (10)

$$P=\frac{TP}{TP+FP}$$ (11)

$$R=\frac{TP}{TP+FN}$$ (12)

Where, TP denotes the count of accurately detected safety helmets, FP represents the count of falsely detected safety helmets, FN signifies the count of undetected safety helmets, P stands for precision rate, R corresponds to recall rate, N denotes the total number of categories, and i signifies the current category. For each individual class, the mean Average Precision (AP) is the area beneath the respective Precision-Recall (PR) curve.

### 4.3 Ablation study

To verify the effectiveness of each component in the proposed safety helmet detection method, based on the original YOLOv5s, we verify the designed lightweight backbone network, upsampling module, channel attention module and positional attention module one by one. The pre-training model provided by the official website is loaded before training, and data augmentations used include mosaic, mixup, random-erasing, color perturbation, scaling, rotation and translation, etc. The experimental results of the designed ablation experiments are shown in Table 1. It should be noted that the experimental data shown in Table 1 are the results obtained by training on GTX 1080Ti.

**Table 1:** Ablation experiments.

DAPT	LB	UM	CM	PM	FPS	AP		mAP
						helmet	head	
					102	0.896	0.833	0.865
✓					102	0.911	0.883	0.897
✓	✓				141	0.915	0.881	0.898
✓	✓	✓			122	0.925	0.902	0.914
✓	✓	✓	✓		116	0.932	0.916	0.924
✓	✓	✓	✓	✓	98	0.948	0.933	0.941

Note that DAPT represents data augmentation and pre-training weights; LB represents the designed lightweight backbone network; UM represents the designed upsampling module; CM represents the designed channel attention module; PM represents the designed positional attention module.

#### (1) Effectiveness of data augmentation and pretrained models

By comparing the experimental data in the second and third rows of Table 1, it can be found that compared with training from scratch, loading the officially provided pre-training model and enabling Mosaic and other data enhancements for training are more effective. In particular, mAP is improved by 3.2%, the AP of head class is improved by 6%. This verifies that it is very necessary to use a pre-trained model and data augmentation for the helmet detection task.

#### (2) Effectiveness of lightweight backbone network

By comparing the experimental data in rows 3 and 4 in Table 1, it can be found that it is effective to use the proposed IR structure to optimize BottleneckCSP in the YOLOv5 backbone network. Although there is little improvement in mAP, the inference speed is 30% faster than the original YOLOv5. This shows that the designed lightweight backbone network can well speed up the inference speed of the detection model.

#### (3) Effectiveness of the upsampling module

By comparing the experimental data in the 4th and 5th rows of Table 1, it can be found that although the FPS has decreased, the mAP has increased by 1.6%. This shows that replacing traditional interpolation methods with subpixel convolution and dilated convolution based upsampling modules is beneficial for safety helmet detection.

#### (4) Effectiveness of channel attention module

Comparing the experimental data of rows 5 and 6 in Table 1, it can be found that the AP of both hat and head are improved, at a small cost of FPS. Overall, the designed channel attention module helps to improve the performance of helmet detection.

#### (5) Effectiveness of positional attention module

By comparing the last two rows of experimental data in Table 1, it can be found that on the basis of the channel attention module, after concatenating a position attention module, the AP of both hat and head has been steadily improved. Specifically, the AP of hat is improved by 1.6%, and the AP of head is improved by 1.7%, which well demonstrates the effectiveness of the designed positional attention module.

### 4.4 Comparison with state of the arts

To verify the superiority of the SHDet proposed in this paper, we compare it with the safety helmet detection methods published in recent years. Table 2 shows the experimental results of our method compared with other safety helmet detection methods on the self-made dataset. The experimental results of the comparative methods in Table 2 are derived from the papers published by the authors themselves.

**Table 2:** Comparison experiments with other safety helmet detection methods was conducted on a self-made dataset.

Methods	GPU type	number of images	data split	input size	mAP	FPS
Ai et al. [22]	Tesla P100	5168	80:0:20	448×448×3	0.819	14
Guang et al. [21]	-	3174	50:0:50	416×416×3	0.881	-
Sun et al. [23]	-	-	-	-	0.874	-
Dai et al. [20]	Tesla V100	4523	75:0:15	448×448×3	0.868	83
Chen et al. [19]	GTX 2080	1065	80:0:20	600×1000×3	0.943	12
Deng et al. [25]	GTX 1660Ti	-	88:0:12	416×416×3	0.929	15
Han et al. [28]	RTX 2080Ti	-	80:4:16	640×640×3	0.922	333
Ours	GTX 1080Ti	6116	80:10:10	640×640×3	0.941	98
Ours	RTX 2080Ti	6116	80:10:10	640×640×3	0.942	416

It is not difficult to see from Table 2 that although the highest mAP is the helmet wearing detection method based on Faster R-CNN proposed by Chen et al. [18], it belongs to a two-stage detection method. When they used a GTX 2080 for inference, the FPS was only 12. This can not meet the real-time requirements, and it is difficult to apply to low-profile embedded devices. When the proposed SHDet method uses GTX 1080Ti inference, the FPS can reach 98, which can meet the real-time requirements. At present, the fastest reasoning speed is the helmet detection method based on YOLOv5 proposed by Han et al [28]. But under the same computing power, the proposed SHDet method is faster than the method proposed by Han et al. Overall, the method proposed in this paper achieves good results in both performance and speed.

Furthermore, to verify the generalization performance of the proposed method, we also compared the SHDet method with the helmet detection methods published in recent years on the public dataset SHWD. The experimental comparison results are shown in Table 3.

**Table 3:** Comparison between SHDet method and other safety helmet detection methods on SHWD dataset.

Methods	GPU type	data split	input size	P	R	mAP	FPS
Ai et al. [22]	RTX 2080Ti	8:1:1	640×640×3	0.705	0.565	0.785	9
Guang et al. [21]	RTX 2080Ti	8:1:1	640×640×3	0.796	0.635	0.851	95
Sun et al. [23]	RTX 2080Ti	8:1:1	640×640×3	0.781	0.659	0.868	112
Dai et al. [20]	RTX 2080Ti	8:1:1	640×640×3	0.772	0.648	0.851	28
Chen et al. [19]	RTX 2080Ti	8:1:1	640×640×3	0.810	0.734	0.919	107
Deng et al. [25]	RTX 2080Ti	8:1:1	640×640×3	0.824	0.718	0.914	42
Han et al. [28]	RTX 2080Ti	8:1:1	640×640×3	0.833	0.672	0.907	333
Ours	RTX 2080Ti	8:1:1	640×640×3	0.825	0.786	0.926	416

To maintain a fair comparison, we diligently reproduced the code based on the methodology outlined in the author’s published paper. This meticulous approach ensured that the experimental evaluation was conducted under identical input resolutions, data distributions, and hardware platforms. As evident from Table 3, it becomes apparent that the proposed SHDet method outperforms alternative approaches in terms of both performance and speed. This outcome serves as additional evidence to substantiate the superiority of our proposed method within the experimental context.

## 5. Conclusion

To make the designed network run on mobile terminals or embedded devices with limited computing power, this paper simplifies the backbone network of YOLOv5, which increases the inference speed by 30%. Experimental results show that the designed upsampling module, channel attention module and positional attention module can be integrated into the detection framework of YOLOv5 to further improve the detection performance of safety helmet. Among them, the designed up-sampling module can enhance the semantic information of the feature map during the reverse transfer process. The designed attention module can enhance the semantic information of shallow feature maps to improve the detection rate of small objects. Cascaded channels and positional attention modules can better fuse deep semantic information and shallow spatial information, enabling the network to extract saliency features. Compared with the helmet detection algorithms proposed in recent years, the overall performance and speed of SHDet are state of the art. The limitation of this paper is that although the accuracy of safety helmet detection is high, it is not optimal, and there is still room for further optimization. To this end, it is planned to introduce self-attention mechanism in Transformer into YOLOv5’s framework in the future to enhance the model’s ability to extract global context features.
